# Invasive Fungal Disease in High-Acuity Hospitals in Latin America: Practical Pathways from Suspicion to Early Targeted Therapy

**DOI:** 10.3390/jof12100770

**Published:** 2026-10-14

**Authors:** Juan David Plata Puyana, Daniela Rico, Alejandro Iregui, Fredy Guevara

**Affiliations:** Fundación Santa Fe de Bogotá, Bogotá 110231, Colombia; daniela.rico@fsfb.org.co (D.R.); eduardo.iregui@fsfb.org.co (A.I.); fredy.guevara@fsfb.org.co (F.G.)

**Keywords:** invasive fungal disease, candidemia, invasive aspergillosis, mucormycosis, cryptococcal meningitis, Latin America, diagnostic pathway, antifungal stewardship, high-acuity hospitals, galactomannan

## Abstract

Invasive fungal diseases (IFDs) have become a routine diagnostic and therapeutic challenge in high-acuity hospitals worldwide, and Latin America presents a distinctive scenario in which opportunistic mycoses of tertiary care coexist with endemic dimorphic fungi, heterogeneous diagnostic capacity, and uneven antifungal access. This narrative review organizes a practical, clinically usable pathway for Latin American high-acuity hospitals, from the construction of a “window of suspicion” based on host risk factors, through the diagnostic work-up available in the region (non-culture biomarkers, molecular platforms, imaging, and histopathology), to syndrome-specific clinical pathways for candidemia, invasive aspergillosis, mucormycosis, and cryptococcal meningitis, including antifungal selection, dosing, and stewardship. We highlight where regional diagnostic and therapeutic constraints depart from guidance developed in high-resource settings and where structured clinical suspicion must substitute for advanced biomarkers. We conclude that early, syndrome-directed pathways adapted to local resource availability, rather than uncritical adoption of international algorithms, offer the most realistic route to earlier antifungal therapy and improved outcomes in Latin American high-acuity hospitals.

## 1. Introduction

Invasive fungal diseases (IFDs) have evolved from occasional complications confined to a narrow group of immunocompromised patients into a daily diagnostic and therapeutic challenge in modern high-acuity hospitals worldwide. Their clinical impact is substantial: they are difficult to recognize early, frequently occur in patients with a high burden of underlying disease, and are associated with prolonged hospitalization, increased costs, and significant mortality. Some estimates suggest that severe fungal diseases account for millions of cases worldwide every year, with invasive candidiasis and invasive aspergillosis representing major contributors to the global burden [1]. Candidemia remains particularly lethal; population-level data indicate that approximately one third of patients die during hospitalization, and invasive candidiasis adds measurable hospital days and costs [2]. Invasive aspergillosis is similarly severe in critical care, where delayed antifungal therapy is associated with longer ICU stay, higher costs, and excess mortality [3]. This burden is increasing as susceptible populations expand: transplantation, oncohematology, biologic immunomodulation, prolonged ICU care, severe viral pneumonias, advanced liver disease, and the post-COVID clinical landscape have all increased the number of patients in whom fungal infection must be considered early rather than as a last diagnostic resort.

In Latin America, the problem is not simply a regional version of the epidemiology described in Europe or North America. Opportunistic mycoses typical of tertiary hospitals coexist with endemic dimorphic fungi, including *Histoplasma* and *Paracoccidioides*, which remain underdiagnosed and unevenly represented in surveillance systems [4,5]. These endemic mycoses are themselves markedly heterogeneous across the region: paracoccidioidomycosis is concentrated in the humid agricultural areas of Brazil, Colombia, and Venezuela, coccidioidomycosis is largely confined to arid regions of Mexico, Brazil, Argentina, Venezuela, and La Guajira, Colombia, and the burden of histoplasmosis tracks the distribution of advanced HIV disease rather than a single ecological niche (Section 2.2). Histoplasmosis illustrates this gap: in advanced HIV, nonspecific presentation, overlap with tuberculosis, and limited access to antigen testing contribute to diagnostic delay and preventable mortality [6]. At the same time, *Candidozyma auris* (*Candida auris*) has emerged across multiple Latin American countries, exposing weaknesses in species-level identification, susceptibility testing, infection prevention, and regional coordination [7]. Mucormycosis has also gained visibility after COVID-19, particularly among patients with diabetes, corticosteroid exposure, and severe viral illness [8]. This diversity means that there is no single “Latin American reality” for IFD management.

Structural underdiagnosis is a central feature of this scenario. Blood cultures have limited sensitivity for invasive candidiasis, conventional cultures are slow, histopathology is not always rapidly available, and biomarkers such as galactomannan, beta-D-glucan, fungal PCR, *Histoplasma* antigen, and therapeutic drug monitoring (TDM) remain inconsistently accessible. A comparative analysis specifically contrasting Brazil with Europe—not necessarily generalizable to other Latin American health systems—showed marked gaps in access to galactomannan, beta-D-glucan, *Aspergillus* PCR, MALDI-TOF identification, echinocandins, liposomal amphotericin B (L-AmB), and azole TDM, underscoring that guideline implementation depends on infrastructure as much as on medical knowledge [9]. Thus, guidelines developed in high-resource settings remain essential but do not fully capture the diagnostic constraints, endemic pathogens, antifungal access barriers, and heterogeneous realities of Latin American hospitals.

The high-acuity hospital is the natural setting in which these tensions converge. Intensive care units, oncology and hematology services, transplant programs, major surgery, invasive devices, broad-spectrum antibacterial exposure, renal replacement therapy, parenteral nutrition, and severely immunocompromised hosts concentrate risk in the same physical and organizational space. These institutions also concentrate the opportunity for intervention, because they can connect bedside suspicion, radiology, microbiology, pathology, source control, stewardship, and early antifungal optimization through explicit pathways.

This narrative review responds to a practical gap: the lack of clinically usable pathways adapted to Latin American high-acuity hospitals. Rather than proposing a meta-analysis or a universal algorithm, its objective is to organize the pathway from clinical suspicion to early directed therapy, integrating host risk, syndrome recognition, local epidemiology, diagnostic availability, and antifungal stewardship. In environments where advanced biomarkers may be unavailable or delayed, structured suspicion becomes the first diagnostic tool. The aim is to help clinicians think earlier, sample better, interpret tests in context, and treat decisively when the probability of IFD becomes clinically meaningful.

Because this review draws on quantitative and practice-oriented data from primary studies, regional surveys, and international guidelines, PubMed/MEDLINE, LILACS, and SciELO were searched for literature published from 2010 onward, using combinations of terms including invasive fungal disease, candidemia, invasive aspergillosis, mucormycosis, cryptococcosis, histoplasmosis, antifungal stewardship, and Latin America together with individual country names; reference lists of retrieved guidelines and reviews were hand-searched for additional sources. Because the underlying literature spans randomized trials, observational cohorts, regional surveillance surveys, and consensus guidelines that are not amenable to a single quality-appraisal framework, we opted for a narrative rather than a systematic synthesis: sources were selected for relevance to the clinical pathway described here rather than through a predefined systematic screening protocol.

## 2. Clinical Suspicion and Host Risk Factors

### 2.1. The Window of Suspicion

The first step in any pathway for invasive fungal disease is not a laboratory test, but the construction of a “window of suspicion.” This window opens when the trajectory of a vulnerable host becomes discordant with the presumed bacterial, viral, inflammatory, or postoperative explanation. IFD often enters the differential diagnosis late because its manifestations are nonspecific: persistent fever, culture-negative sepsis, respiratory deterioration, organ dysfunction, or radiologic abnormalities that mimic bacterial pneumonia, tuberculosis, embolic disease, malignancy, or inflammation. In the ICU, this ambiguity is intensified by sedation, ventilation, broad-spectrum antibiotics, devices, and overlapping causes of shock. The pathway should therefore define when the clinician must stop asking only “why is the patient not improving?” and start asking “could this be fungal disease?”

Host risk factors can be organized into four practical categories. The first is classic immunosuppression. Prolonged neutropenia remains a major risk factor for mold disease and is incorporated into international definitions when neutrophil counts fall below 500 cells/µL for more than 10 days [10]. Allogeneic hematopoietic stem cell transplantation (HSCT), solid organ transplantation (SOT), hematologic malignancy, graft-versus-host disease (GVHD), sustained corticosteroids, and T-cell or B-cell immunosuppressants are also core host factors; the EORTC/MSGERC threshold for corticosteroids is at least 0.3 mg/kg/day prednisone equivalent for three or more weeks [10]. Biologic agents and targeted immunomodulators add further complexity, including anti-TNF, IL-17 pathway inhibitors, and JAK inhibitors.

The second category is ICU-acquired vulnerability. Many critically ill patients who develop IFD do not meet classical definitions designed for neutropenic or transplant populations. Mechanical ventilation, central venous catheters, parenteral nutrition, renal replacement therapy, broad-spectrum antibacterials, abdominal surgery, pancreatitis, anastomotic leak, and shock create a risk ecology in which *Candida* and *Aspergillus* may become clinically relevant without traditional immunosuppression. FUNDICU was developed because non-neutropenic ICU patients often fall outside classical frameworks, particularly in severe influenza, COVID-19, cirrhosis, COPD, solid tumors, and uncontrolled HIV [11]. For *Candida*, the IDSA guideline recommends considering empiric antifungal therapy in critically ill patients with risk factors and no other fever source, especially in septic shock [12]. This recommendation must be read together with the negative results of a subsequent placebo-controlled trial in this same population, discussed in Section 5.3: the presence of risk factors alone should prompt diagnostic escalation, not automatically trigger empirical therapy.

The third category is underlying disease that points toward specific fungal syndromes. Diabetes mellitus, especially when uncontrolled or accompanied by ketoacidosis, should raise concern for mucormycosis in compatible rhino-orbital, cerebral, pulmonary, or cutaneous disease. Cirrhosis increases susceptibility to candidemia through immune dysfunction, gastrointestinal translocation, invasive care, and frequent hospitalization. Advanced HIV remains a central risk factor for cryptococcosis and disseminated histoplasmosis in Latin America, where histoplasmosis is often misdiagnosed as tuberculosis [6]. Severe COPD, structural lung disease, lung cancer, and corticosteroids should increase suspicion for aspergillosis when deterioration is unexplained.

The fourth category includes emerging and contextual risks. Severe COVID-19 introduced ICU fungal syndromes such as COVID-19-associated pulmonary aspergillosis (CAPA) and mucormycosis. CAPA is difficult to diagnose because many patients lack classic host factors, imaging may be nonspecific, bronchoscopy may be delayed, and serum galactomannan has limited sensitivity [13]. Prior echinocandin exposure, recurrent healthcare contact, prolonged hospitalization, and local outbreaks should raise concern for *C. auris*, for which rapid species-level identification remains limited in the region [7]. Colombian surveillance has shown ongoing transmission with clinically relevant azole and amphotericin B resistance [14].

Clinical alarm signs should be interpreted as syndrome-host combinations rather than isolated findings. Persistent fever despite broad-spectrum antibiotics is the classic trigger, but the more useful signal is the pattern: fever without bacterial source in a colonized ICU patient; recurrent fever after severe COVID-19; progressive pulmonary infiltrates during corticosteroids; septic shock after abdominal surgery with negative bacterial cultures; or neurological symptoms in advanced HIV or transplant recipients. Nodules, cavitation, the halo sign, airway lesions, or progressive ground-glass abnormalities should prompt consideration of invasive mold disease. The IDSA aspergillosis guideline recommends chest CT whenever invasive pulmonary aspergillosis (IA) is suspected and supports bronchoalveolar lavage (BAL) when feasible [15].

**Figure 1:**
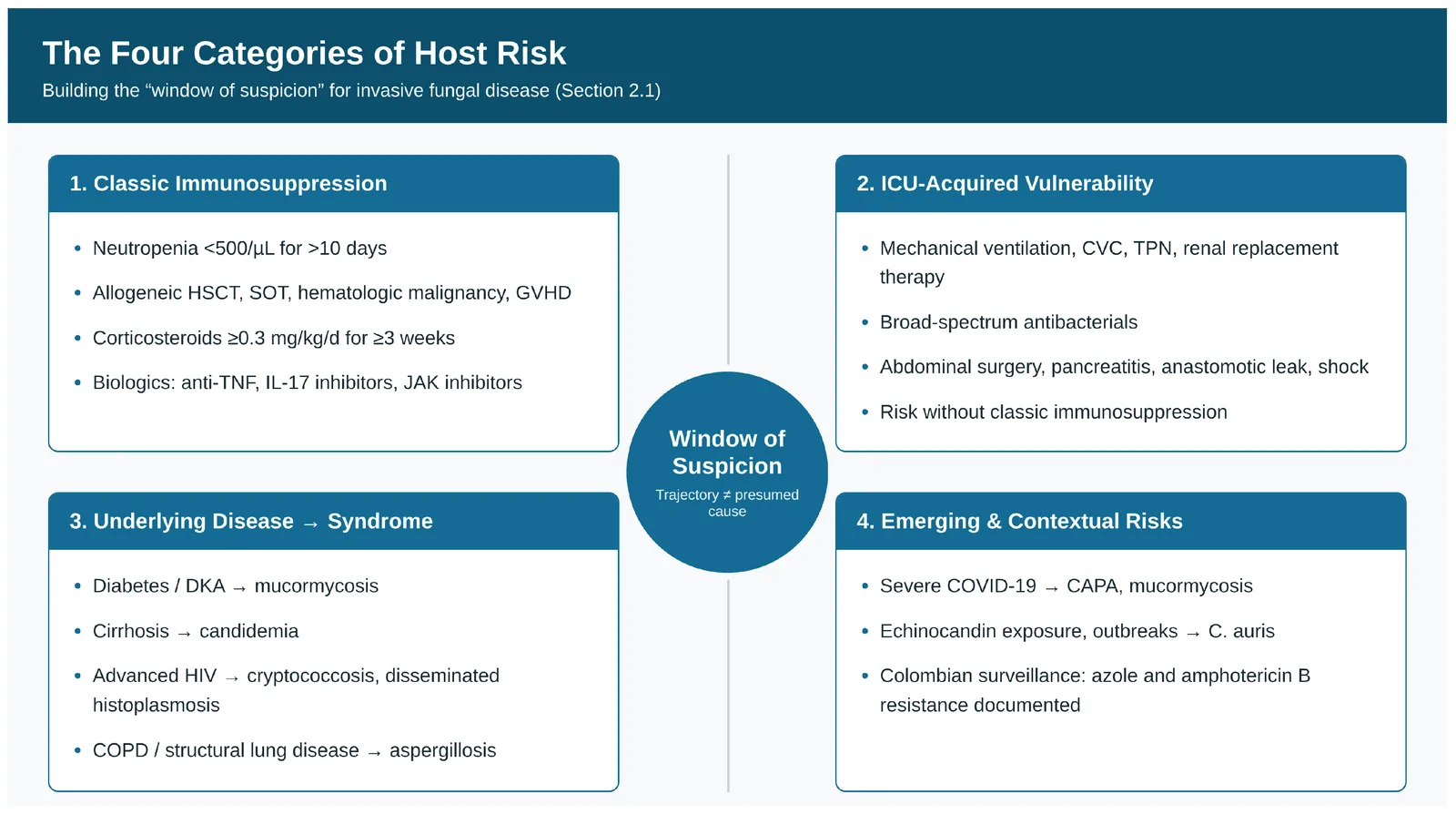
The four categories of host risk that build the “window of suspicion” for invasive fungal disease: classic immunosuppression, ICU-acquired vulnerability, underlying disease, and emerging/contextual risks (Section 2.1).

Skin, mucosal, neurological, and rhino-orbital findings can shorten the pathway. Disseminated skin lesions in advanced HIV should prompt evaluation for histoplasmosis and other disseminated mycoses. Headache, altered mental status, cranial nerve abnormalities, or meningeal symptoms should trigger urgent cryptococcal evaluation in advanced HIV. Facial pain, orbital swelling, black eschar, palatal necrosis, or rapidly progressive necrosis in a patient with diabetes, COVID-19, or corticosteroid exposure should be treated as possible mucormycosis. Section 2.2, Section 2.3, Section 2.4 and Section 2.5 detail these risk factors, syndromes, and alarm findings organized by pathogen (Figure 1).

### 2.2. Pathogen-Specific Risk Factors

Early identification of IFD requires integrating host factors, immunosuppression intensity, clinical syndrome, and diagnostic tools (Table 1).

Candidemia and invasive candidiasis are frequent in the ICU and are driven by cumulative risk from antibiotic exposure, central venous catheters, and high-risk abdominal surgery (Table 1; see also Section 5.1 for detailed odds ratios and Table 2 for risk factors).

Invasive aspergillosis is associated with profound (<500 cells/µL) and prolonged (>10 days) neutropenia, allogeneic transplantation, GVHD, and corticosteroids (≥0.3 mg/kg/day for ≥3 weeks). It should also be considered in critically ill patients with epithelial injury caused by influenza or SARS-CoV-2 (IAPA/CAPA), especially in the setting of mechanical ventilation, COPD, or cirrhosis (Table 1; see also Table 3 and Table 4 and Section 5.2 for host-risk stratification).

Mucormycosis: diabetic ketoacidosis remains one of the dominant triggers, particularly in rhino-orbito-cerebral disease, alongside classic hematologic and post-COVID corticosteroid-related pathways (Table 1).

Cryptococcosis reflects profoundly impaired cell-mediated immunity; the main global risk factor is advanced HIV infection (CD4+ < 100/µL), followed by solid organ transplantation, hematologic malignancies, and anti-CD20 therapies (Table 1).

Endemic mycoses in Latin America are shaped by ecological niches: *Histoplasma capsulatum* in bird/bat guano and caves, *Paracoccidioides* spp. in humid agricultural areas of Brazil, Colombia, and Venezuela, and *Coccidioides* spp. in arid regions of Mexico, Brazil, Argentina, Venezuela, and La Guajira, Colombia. Disseminated histoplasmosis is especially relevant with CD4+ < 200/µL (Table 1).

### 2.3. Special Populations and Risk Stratification

The categories introduced in Section 2.1 (Figure 1) translate into distinct risk profiles across specific patient populations, summarized below.

Hematology–oncology: AML and MDS receiving intensive chemotherapy carry the highest risk (10–25%), driven by >10–14 days of neutropenia and mucosal injury. Relapsed/refractory hemopathies significantly increase mold infection risk (HR 3.43), predominantly *Aspergillus* spp. and *Mucorales*. ALL poses an intermediate-high risk (6.5–15%), driven by induction corticosteroids. Baseline risk in lymphomas, CLL, and multiple myeloma is low (<3%), but increases markedly with targeted therapies: ibrutinib is associated with early aspergillosis with CNS involvement (up to 40%); venetoclax plus hypomethylating agents are associated with IFD rates of 8–13%; alemtuzumab/fludarabine favor *Pneumocystis jirovecii*, cryptococcosis, and mucormycosis; and CAR-T therapy is associated with an IFD incidence of ~3%, escalating with prolonged neutropenia, lymphopenia, inflammatory toxicity, or corticosteroids.

HSCT: risk concentrates in allogeneic transplants (autologous < 3%), peaking with active leukemia, refractory disease, or prior IFD. Pre-engraftment features neutropenia, mucositis, catheters, and candidiasis. Post-engraftment, GVHD (grades II–IV), corticosteroids, and antithymocyte globulin strongly increase *Aspergillus* spp. and *Mucorales* risk. Critical risk modifiers include haploidentical transplantation (HR 2.41); specifically in allo-HSCT, acute GVHD (HR 3.23) and chronic GVHD (HR 2.95) independently increase mold infection risk, while CMV disease specifically raises aspergillosis risk (HR 3.23).

SOT follows a classic chronology: within 1 month, *Candida* spp. predominates, driven by surgical complications. From 1–6 months, *Aspergillus* spp. and other opportunists rise, driven by immunosuppression, CMV (OR 2.97), and lung-transplant factors. After >6 months, *Cryptococcus* spp. and endemic mycoses emerge, often reactivated by rejection or intensified immunosuppression.

ICU: two high-risk phenotypes predominate: complicated intra-abdominal infection with candidiasis/candidemia, associated with 40–60% mortality when source control is delayed; and severe viral ARDS complicated by IAPA/CAPA, reported in 10–20% of critically ill influenza or COVID-19 patients, with mortality around 40–60%.

HIV/AIDS: disseminated histoplasmosis requires strong suspicion at CD4+ < 200 cells/µL (especially <150 cells/µL) presenting with fever, cytopenias, or respiratory involvement; it frequently mimics tuberculosis, delaying diagnosis. Cryptococcal meningitis remains a lethal threat, classically associated with CD4+ < 100 cells/µL.

### 2.4. Scores and Risk Stratification Tools

Predictive rules complement clinical judgment primarily by excluding disease through their high negative predictive value (NPV), which depends on pretest prevalence; they are not meant to replace bedside decision-making. The *Candida* Score—severe sepsis, surgery, parenteral nutrition, and multifocal colonization—with a cutoff ≥3 (sensitivity 81%, specificity 74%) was originally designed to guide empirical therapy initiation [16]; however, its low positive predictive value (PPV, ~10–15%) limits this use in practice, and contemporary stewardship-oriented literature therefore groups it with other rules as predominantly useful for excluding low-risk patients rather than triggering treatment [17]. The Ostrosky-Zeichner rule combines central venous catheters or broad-spectrum antibiotics with minor factors (surgery, parenteral nutrition, dialysis, immunosuppression), providing excellent specificity (~83%) and NPV (97–99%), but low sensitivity (~50%) and PPV (~10%) [18]. To overcome low PPV, the Nebraska rule refines selection using abdominal surgery and prior ICU stay (NPV 99.4%) [19]. Coupling these scores with biomarkers (e.g., β-D-glucan) increases specificity and PPV while maintaining NPV >99%, reducing unnecessary echinocandin use.

**Figure 2:**
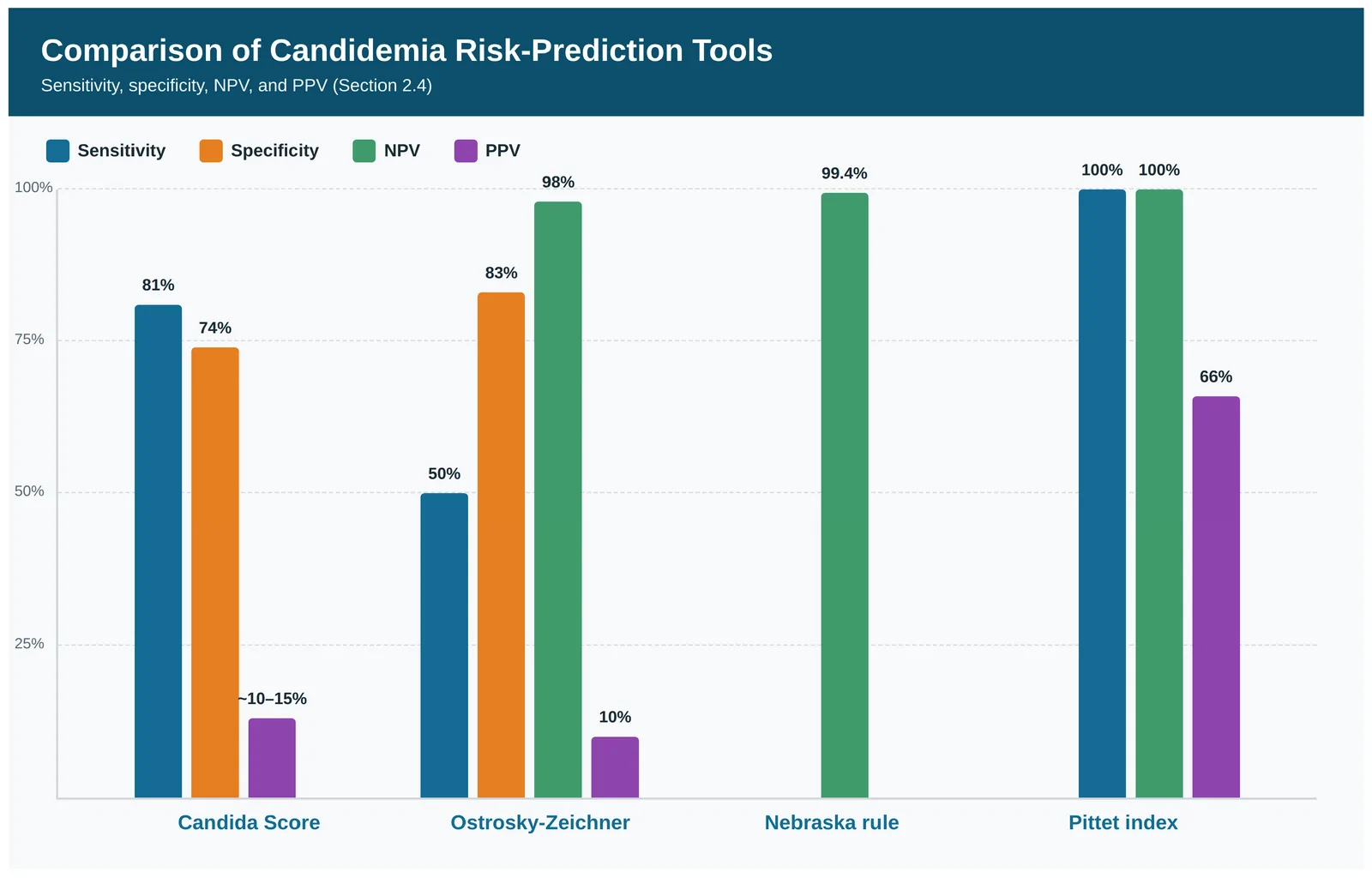
Comparison of candidemia risk-prediction tools—*Candida* Score, Ostrosky-Zeichner rule, Nebraska rule, and Pittet colonization index—by sensitivity, specificity, NPV, and PPV. Dashed boxes indicate values not reported in the cited literature (Section 2.4).

The classic Pittet colonization index (cutoff ≥0.5) offers strong prediction (sensitivity 100%, NPV 100%, PPV ~66%) but requires impractical serial surveillance cultures and is only useful if surveillance is standardized [20]. The EORTC/MSGERC criteria do not dictate empirical treatment: they classify IFDs as proven, probable, or possible for research purposes in classic hosts (hematology–oncology, HSCT, SOT) [10]. In the general ICU, adapted algorithms (AspICU, FUNDICU) that integrate clinical features, biomarkers, and microbiology should be used [11,21]. In Latin America, scores require caution because biomarker access is limited, surveillance is not routine, species distribution differs, endemic mycoses enter the differential diagnosis, and antifungal therapy may be constrained. A regional pathway should use scores as guardrails, not gates. The infectologist’s role is to integrate local epidemiology, host phenotype, trajectory, diagnostic feasibility, and therapeutic consequences. The key question is whether the patient’s course justifies activating a fungal diagnostic bundle and considering early antifungal therapy while data are pending (Figure 2). It is worth emphasizing that clinical suspicion, by itself, is the trigger for this diagnostic escalation, not for treatment. As developed in Section 5.3, the decision to start empirical, pre-emptive, or targeted antifungal therapy requires this diagnostic bundle and, where feasible, a biomarker- or imaging-supported estimate of probability—layered on top of host risk and clinical trajectory, rather than suspicion alone.

### 2.5. Clinical and Radiologic Red Flags

Given delayed microbiological confirmation, red flags must trigger early evaluation and therapy. A key warning sign is persistent fever after 3–7 days of broad-spectrum antibiotics in neutropenic or critically ill patients. Multiple erythematous-violaceous papulonodular skin lesions, often appearing during neutrophil recovery, suggest hematogenous candidiasis. On chest CT, nodules and the halo sign suggest angioinvasive aspergillosis; conversely, a reverse halo, multiple nodules (>10), or pleural effusion point toward pulmonary mucormycosis in diabetic or immunosuppressed patients. In this context, facial pain, proptosis, or periorbital edema require urgent MRI; the black turbinate sign indicates early mucormycosis ischemia. Painful necrotic skin lesions or black eschars suggest angioinvasive mold dissemination. In endemic areas, hilar/mediastinal lymphadenopathy with reticulonodular infiltrates suggests pulmonary histoplasmosis. In advanced HIV infection, fever, cytopenias, hepatosplenomegaly, and progressive lymphadenopathy indicate disseminated disease. Table 1 consolidates host risk factors, clinical/radiological red flags, and first-line diagnostic actions by pathogen for rapid bedside use.

## 3. Epidemiology

Regional epidemiologic data for IFDs remain limited, coming mainly from a small number of multicenter, laboratory-based surveillance networks rather than national notification systems [22]. Nevertheless, they consistently show a pattern distinct from high-resource settings in incidence, age distribution, and species mix, reinforcing the case for locally adapted pathways rather than direct extrapolation from international guidelines.

Candidemia is the best-characterized syndrome in the region. A 24-month, laboratory-based survey across 21 tertiary hospitals in seven Latin American countries documented an overall incidence of 1.18 cases per 1000 hospital admissions, with the highest rates in Colombia and a notably high pediatric share (44.2% of episodes) compared with North America or Europe [23]. *Candida albicans* remained the leading agent (37.6%), followed by *C. parapsilosis* (26.5%) and C. tropicalis (17.6%), with marked country-to-country variability and, in some countries, a high local prevalence of *C. guilliermondii* [23], consistent with earlier regional reviews of a comparatively high non-albicans *Candida* burden [4].

Invasive aspergillosis is far less systematically documented in the region; most series originate from single hematology–oncology or transplant centers rather than multicenter surveillance. *Aspergillus* is consistently the leading mold pathogen in allogeneic HSCT recipients, with a bimodal risk distribution around engraftment and later immunosuppression [22]; population-level incidence and mortality estimates remain largely extrapolated from global models rather than measured directly.

Mucormycosis has historically been considered rare outside of hematologic and diabetic populations, but its regional visibility increased sharply after the COVID-19 pandemic, with case clusters reported across several Latin American countries in patients with diabetes, corticosteroid exposure, and severe viral pneumonia [8].

Cryptococcal disease remains a leading cause of HIV-associated mortality in the region. Global burden-of-disease modeling has estimated more than 5000 incident cases of HIV-associated cryptococcal meningitis annually in Latin America and the Caribbean, with approximately 2400 attributable deaths per year, placing the region among those with a substantial absolute burden outside sub-Saharan Africa [24]. Reported case-fatality rates from Latin American cohorts are correspondingly high, in the order of 30–63% depending on the setting and on access to timely diagnosis and induction therapy.

Among the endemic mycoses, histoplasmosis is a particularly consequential and under-recognized disease in people with advanced HIV: modeling suggests an incidence comparable to tuberculosis in the region, with histoplasmosis mortality meeting or exceeding tuberculosis mortality in several countries despite receiving far less diagnostic and programmatic attention [25]. Paracoccidioidomycosis and coccidioidomycosis remain confined to the ecological niches described in Section 2.2, with incidence data derived mainly from country-level case series rather than systematic surveillance [5].

Finally, the regional emergence of *C. auris* since the mid-2010s has added a resistance-associated dimension to the region’s candidemia burden, with documented in-hospital outbreaks and, in Colombia, genomic surveillance showing ongoing transmission with clinically relevant azole and amphotericin B resistance [7,14].

## 4. Diagnostic Pathway: From Suspicion to Confirmation

**Figure 3:**
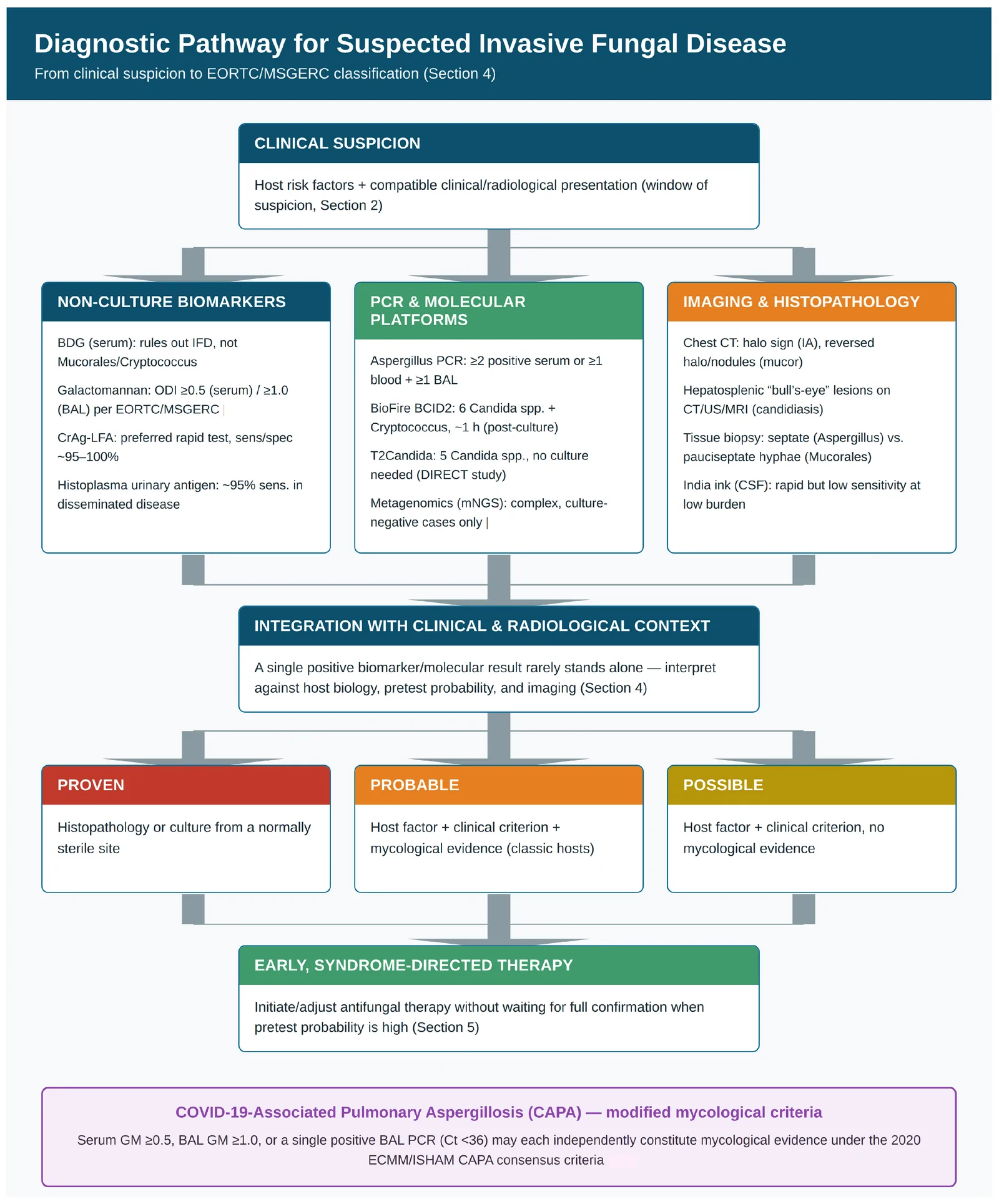
Diagnostic pathway for suspected invasive fungal disease (IFD), from clinical suspicion and targeted mycological work-up to classification under the EORTC/MSGERC definitions. HSCT, hematopoietic stem-cell transplant; SOT, solid-organ transplant; BAL, bronchoalveolar lavage; GM, galactomannan; BDG, (1,3)-β-D-glucan; CrAg-LFA, cryptococcal antigen lateral-flow assay; CSF, cerebrospinal fluid; CT, computed tomography (Section 4).

The diagnosis of IFD integrates host factors, clinical–radiological findings, and mycological evidence. The EORTC/MSGERC definitions classify disease as proven, probable, or possible (Figure 3). In Latin America, diagnosis must be adapted to available resources without delaying treatment when clinical suspicion is high. Throughout this section, the sensitivity and specificity figures reported for each biomarker should be interpreted in light of the patient’s pretest probability, the host population in which the test was validated, and local test availability—a single cut-off or performance figure should not be extrapolated uniformly across hosts or settings (Section 2.4).

### 4.1. Non-Culture-Based Biomarkers

(1,3)-β-D-glucan (BDG). This is a fungal biomarker detectable in *Candida*, *Aspergillus*, and *Pneumocystis jirovecii* infections (absent in *Mucorales* and *Cryptococcus* spp.). Its greatest value is to rule out IFD through its high negative predictive value, which depends on pretest prevalence; it does not confirm the diagnosis on its own, and specificity improves with two positive tests [10,26]. In meta-analyses, sensitivity is 77–80% and specificity 82–85%, with variation according to population and assay [26,27]. False positives are associated with immunoglobulins, albumin, some hemodialysis membranes, and surgical gauze, and sensitivity decreases under antifungal prophylaxis. Manufacturer cutoffs are ≥80 pg/mL for Fungitell^®^ and ≥11 pg/mL for Wako^®^ [28].

Galactomannan (GM). In the Platelia™ assay, the EORTC/MSGERC definitions accept as mycological evidence an optical density index (ODI) ≥ 1.0 in serum/plasma, BAL, or CSF, or the combination of serum/plasma ≥ 0.7 and BAL ≥ 0.8 [10]. Its performance depends on the cutoff and the sample: in serum, at ODI ≥ 0.5 sensitivity is 78% and specificity 85%, and at ≥1.0 sensitivity falls (71%) but specificity rises (90%) [29]; BAL is more sensitive than serum, performance is higher in neutropenic patients, and decreases with active mold-directed prophylaxis [29,30]. For CAPA, the ECMM/ISHAM criteria accept serum GM ≥ 0.5 or BAL GM ≥ 1.0 [13]. Rapid lateral-flow tests (LFA/LFD) are alternatives when ELISA is unavailable, with results not necessarily interchangeable between platforms [31]. Cross-reactivity with other fungi, including *Fusarium* and *Histoplasma*, should be considered [32].

*Cryptococcus* and *Histoplasma* antigens. The cryptococcal lateral-flow antigen (CrAg-LFA) is the preferred rapid test—with sensitivity and specificity of approximately 95–100%—and can be performed on CSF, serum, plasma, or whole blood [33]; at very high titers, the prozone phenomenon should be excluded [34]. When meningitis is suspected, lumbar puncture remains essential, both for diagnosis and to measure opening pressure [33]. In histoplasmosis, urinary antigen detection—complemented with serum—performs best in disseminated disease, especially in people with HIV, with a pooled sensitivity close to 95% (specificity 97%) that decreases in localized forms [35,36,37].

### 4.2. PCR and Molecular Platforms

*Aspergillus* PCR is incorporated into the EORTC/MSGERC definitions: it requires ≥2 consecutive positive samples in serum/plasma/whole blood, ≥2 positive replicates in BAL, or ≥1 positive in blood plus ≥1 in BAL [10,38]. For CAPA, a single positive BAL PCR with Ct < 36 may also constitute mycological evidence [13]. A positive respiratory result must be interpreted within the clinical–radiological context, as it does not by itself demonstrate tissue invasion [38]. Some platforms, such as AsperGenius^®^, additionally detect CYP51A mutations related to azole resistance [39].

Multiplex panels speed up identification but with limited fungal coverage: BioFire BCID2 detects six *Candida* species—including *C. auris*—and *Cryptococcus neoformans/gattii* in approximately 1 h, although it requires a prior positive blood culture [40]. T2Candida detects five *Candida* species directly in whole blood by T2 magnetic resonance, without culture; in the DIRECT study it achieved a sensitivity of 91.1% and a specificity of 99.4%—part of the validation was performed with spiked samples [41]. It does not include *C. auris* or replace culture for susceptibility; T2 Biosystems ceased operations in 2026, limiting its future availability [42]. Metagenomics (mNGS) is reserved for complex cases with a negative conventional work-up and remains concentrated in reference centers [43].

### 4.3. Imaging

When invasive pulmonary aspergillosis is suspected, a chest CT should be requested early, regardless of conventional radiography findings; the halo sign is especially suggestive of angioinvasive aspergillosis in the neutropenic patient [15]. The reversed halo, pleural effusion, and the presence of multiple nodules increase the suspicion of mucormycosis, although none of these findings is specific [44]. In hepatosplenic candidiasis, CT, ultrasound, or MRI show “bull’s-eye” hepatosplenic lesions, most evident during recovery from neutropenia [12].

### 4.4. Culture, Microscopy, and Histopathology

Blood cultures remain the conventional reference method for candidemia, but they have limited sensitivity in invasive candidiasis, especially in deep-seated disease without fungemia [45]. When pulmonary aspergillosis is suspected, BAL should be sent for direct examination, culture, GM, and, when available, PCR; the presence of septate hyphae with acute-angle branching is compatible with *Aspergillus*, but is not genus-specific [15].

In mucormycosis, tissue biopsy is essential: histopathology typically shows broad, irregular, pauciseptate or aseptate hyphae, with a variable branching pattern—frequently wide-angle—necrosis, and angioinvasion. The branching angle can be distorted in tissue, so the sample should also be sent for culture and molecular identification [46]. In central nervous system cryptococcosis, India ink allows a rapid evaluation but loses sensitivity when the fungal burden is low; CrAg-LFA in CSF is the preferred rapid test and should be complemented with culture when possible [33].

## 5. Clinical Pathways

### 5.1. Candidemia

Candidemia is the most frequent form of invasive *Candida* infection, frequently associated with critically ill patients and/or immunosuppressed hosts such as SOT recipients or those with febrile neutropenia. Diagnosis requires a high index of suspicion, as its clinical presentation is nonspecific and clinicians rely on imperfect methods—blood cultures and serum biomarkers—whose results are difficult to interpret in a population at risk for several concomitant nosocomial infections. For Latin America specifically, a recent expert consensus on the diagnosis of candidemia in critically ill patients was published in 2024 [47].

Suspicion for invasive candidiasis typically arises from fever and/or septic shock in a patient with established risk factors (Table 2) [48].

**Table 2:** Risk factors for invasive candidiasis in critically ill patients, adapted from a systematic review and meta-analysis [48] (Section 5.1).

Risk Factors for Invasive Candidiasis in Critically Ill Patients
Broad-spectrum antibiotic exposure—OR 5.6 (95% CI 3.6–8.8)
Blood transfusion—OR 4.9 (95% CI 1.5–16.3)
*Candida* colonization—OR 4.7 (95% CI 1.6–14.3)
Central venous catheter—OR 4.7 (95% CI 2.7–8.1)
Total parenteral nutrition—OR 4.6 (95% CI 3.3–6.3)

Several attempts have been made at external validation of clinical scores such as the Candida Score [16] to identify patients who would benefit from earlier antifungal therapy. Nevertheless, its implementation has been hindered by very low PPV and poor generalizability outside the original derivation cohorts [19]. These scores are more useful as a rule-out tool, particularly when paired with available serological markers [49].

Blood cultures remain the cornerstone of candidemia diagnosis in most Latin American countries. At least two sets (four bottles total), drawn sequentially from different sites, are recommended [47], though sensitivity is poor, with historical figures reported between 21–71% [45]. Newer techniques such as T2Candida and Platelia Candida Ag (mannan antigen, MAg) improve on this performance, but neither is routinely available in the region.

Early recognition of candidemia in the region therefore relies mainly on imperfect clinical scores and blood cultures.

### 5.2. Invasive Aspergillosis

Invasive aspergillosis is largely restricted to severely immunocompromised patients, such as those with SOT and HSCT, but it is also reported in a variety of immunosuppressed patients, such as those with COVID-19, autoimmune disease on DMARDs, cirrhosis, and COPD. Most of the available data regarding clinical presentation, diagnosis, and treatment come from HSCT cohorts, where it is much more prevalent, especially in allogeneic HSCT, where the risk of IA approaches 10%.

Attempts at homogenizing the diagnosis of IA are therefore difficult due to differences in epidemiology, level of immunosuppression, the multiple clinically relevant *Aspergillus* species, and the performance of serum-based diagnostic methods, which may vary across different populations. For Latin America specifically, a companion expert consensus to the candidemia evidence-based clinical standard (EBCS) was published in 2025 on the diagnosis and treatment of invasive lung aspergillosis [50].

Suspicion of IA is first raised in a patient with persistent fever beyond what would be expected despite broad-spectrum antibiotics (febrile neutropenia), or new clinical symptoms or deterioration in patients with established risk factors. Not every type of immunosuppression confers the same risk of developing IA, and although IA can affect any organ, the lungs are the most commonly involved (Table 3 and Table 4) [51,52,53].

**Table 3:** Risk stratification for invasive aspergillosis in classical hosts (Section 5.2).

Risk Stratification for Invasive Aspergillosis (Classical Hosts)	High	Intermediate	Low
Category	AML (first induction); allogeneic HSCT; lung >> heart > liver	ALL; CLL; lymphoma	Autologous HSCT; CML; kidney

**Table 4:** Risk factors for invasive aspergillosis in non-classical hosts (Section 5.2).

Risk Factors for Invasive Aspergillosis (Non-Classical Hosts)
T-cell immunosuppressants
Corticosteroid exposure >700 mg cumulative prednisone-equivalent—OR 2.99–4.07 [51]
Antibiotic exposure before ICU admission in COPD patients—OR 8.0–13.4 [52]
Influenza infection—aOR 5.19 [53]

Definitive diagnosis of IA requires direct visualization of branching septate hyphae in tissue, although this approach may not be feasible in an already compromised, thrombocytopenic, or coagulopathic patient. Cultures (sputum, BAL) are unreliable and difficult to interpret, as *Aspergillus* spp. may be a colonizing organism, especially in lung-transplant patients, among whom cohort data report colonization in up to 30% [54]. Currently, diagnosis depends on a combination of clinical, radiological, and serum-based methods.

A now well-known classification for IA in “classical hosts” was published by the EORTC/MSGERC [55]; originally developed to standardize case definitions for clinical trials and epidemiological research, it is now used at the bedside without external validation in heterogeneous populations. As the lungs are the most commonly involved organ, a high-resolution chest CT is usually the next step, combined with serum biomarkers such as β-D-glucan and galactomannan, and PCR where available.

The performance of serum-based diagnostic methods, as mentioned earlier, is quite variable, and not every population at risk for IA has established performance figures to guide clinical decisions. The most common available methods are the Platelia Aspergillus Ag, Fungitell β-D-glucan, and *Aspergillus* PCR, with variable availability across Latin America. BDG and galactomannan are commercially available in Chile and Mexico. Galactomannan has regulatory approval in Brazil, though BDG use there remains restricted by low availability. BDG has been explicitly reported as unavailable in public hospitals in Buenos Aires, Argentina. A Honduran capacity survey found antigen testing available in only 2.6% of institutions, with none offering PCR.

Cut-off points for galactomannan and β-D-glucan remain a subject of debate; most data are derived from HSCT/hematologic malignancy cohorts, with less well-described performance in SOT or “non-classical” hosts. We advise the reader to interpret these numbers with caution, as host biology differs and using a single cut-off for every suspected case may result in under- or over-diagnosis. Table 5 reports performance figures across host types to the best of our knowledge [56,57,58,59,60,61,62].

### 5.3. Antifungal Therapy: From Empirical to Early Targeted Treatment

The choice of empiric antifungal therapy depends mostly on host risk factors for a specific type of fungal infection, available medications, pharmacokinetic/pharmacodynamic (PK/PD) parameters, and safety profile. Just as the performance of serum-based diagnostics does not apply uniformly to all types of patients, the same is true for antifungal therapy. Historically, critically ill patients with risk factors were candidates for empirical antifungal therapy until the EMPIRICUS trial, published in 2016 [63], compared micafungin with placebo in critically ill non-neutropenic patients and found no significant benefit of empirical antifungal therapy over placebo.

Reconciling this negative trial with the risk-factor-based recommendation noted in Section 2 requires individualizing the decision by host characteristics, disease severity, adequacy of source control, local epidemiology, and the estimated pretest probability of invasive candidiasis, rather than by the presence of risk factors alone. Nevertheless, ICU patients are a heterogeneous population, and some subsets may benefit from early therapy, such as those with upper GI perforations, new-onset septic shock in a previously stable critically ill patient, or settings where local epidemiology reports invasive candidiasis incidences >5–10%.

It is in this situation that the cautious use of the previously mentioned clinical scores is useful to rule out patients who would not benefit from empirical antifungal therapy. Echinocandins such as caspofungin, micafungin, and anidulafungin are guideline-recommended as first-line empirical therapy in this population, though observational data comparing the three agents directly show no clear superiority among them [64,65].

Other populations, such as those with prolonged, profound febrile neutropenia, require a different approach, with more liberal use of empirical antifungals given the high mortality of untreated invasive fungal infections. The distribution of fungal infections in this population tends to be more mold-associated, such as *Aspergillus* and *Mucorales*; therefore, mold-active antifungal therapy is preferred in these clinical situations. The IDSA guideline from 2010/2011 [66] recommends empirical antifungal therapy in patients with persistent or recurrent fever after 4–7 days of antibiotics whose overall expected duration of neutropenia is >7 days. Newer azoles such as voriconazole and isavuconazole have become first-line therapy for suspected IA.

Several mold-active agents are currently available in the region, especially newer triazoles such as posaconazole, voriconazole, and isavuconazole, and polyenes such as amphotericin B and its different formulations. While SOT recipients have their own evidence-based guidelines for the treatment of IA, recommendations are mainly extrapolated from small observational cohorts or the HSCT population, which does not necessarily account for the significant drug–drug interactions encountered with prolonged use of calcineurin inhibitors/mTOR inhibitors in the SOT population.

Two pivotal trials published in the last decade have established voriconazole as the standard of treatment, with posaconazole and isavuconazole as non-inferior second choices for IA. The SECURE trial, published in 2016 [67], was a phase 3 trial comparing voriconazole with isavuconazole in patients with suspected invasive mould disease, stratified by allogeneic HSCT and active malignancy status; notably, SOT recipients were not included. Isavuconazole met the non-inferiority criterion for the 42-day all-cause mortality endpoint, with a better safety profile and fewer treatment discontinuations. A similarly designed phase 3 trial of posaconazole versus voriconazole, published in 2021, again excluded SOT recipients; posaconazole met the non-inferiority criterion for the 42-day all-cause mortality endpoint [68].

Culture-based identification of some fungal organisms is difficult and time-consuming, which limits de-escalation strategies, especially in the treatment of IA; there are no randomized data to guide the duration of IA therapy, and current practice is mainly based on observational studies in heterogeneous populations. Robust data support early de-escalation in candidemia, where yeast readily grow in non-selective media and EUCAST/CLSI breakpoints are available for most clinically relevant yeasts and are frequently included in automated minimum inhibitory concentration (MIC) panels. RCT data published in 2014 [69] support IDSA recommendations for oral step-down to fluconazole or voriconazole after an initial 5 days of IV echinocandin therapy, with similar findings in a more recent observational study supporting less than 5 days of intravenous therapy before de-escalation [70].

As mentioned previously, triazoles have significant drug interactions and safety concerns; most require TDM and frequent follow-up to address adherence and tolerance. It is not uncommon to have to stop azole treatment due to GI intolerance, reducing available options and optimal results. Table 6 reports the general properties of the most commonly used azoles in the region.

There has been increasing interest in antifungal stewardship (AFS) strategies, especially early oral de-escalation and TDM. Efforts have been limited in the region due to the lack of availability of TDM and rapid identification techniques, with a recent paper identifying lack of structure and resources as the main barriers to implementing an effective fungal AFS program [71]. Section 6 expands on these regional antifungal stewardship barriers and opportunities.

### 5.4. Mucormycosis—Medical-Surgical Emergency

Suspicion: compatible host with necrotizing sinusitis, facial edema, proptosis, nasal/palatal eschar, necrotic skin lesions, or atypical pulmonary imaging (Table 1).

Urgent diagnosis: CT for extension/bone involvement, MRI if rhino-orbito-cerebral disease is suspected, and early biopsy for histopathology/culture.

Initial treatment: L-AmB 5 mg/kg/day, or 10 mg/kg/day with CNS involvement, plus urgent repeated surgical debridement. If unavailable, use amphotericin B lipid complex (ABLC) 5 mg/kg/day; avoid deoxycholate when lipid formulations exist.

Consolidation/alternative: isavuconazole 200 mg every 8 h for 6 doses, then 200 mg/day; or posaconazole 300 mg every 12 h on day 1, then 300 mg/day; duration individualized (Table 6). Control of the predisposing factor is essential: correct ketoacidosis/hyperglycemia, reduce immunosuppression when feasible, and discontinue deferoxamine [46].

### 5.5. Cryptococcal Meningitis

Suspicion: subacute headache, fever, visual disturbance, altered mental status, or elevated intracranial pressure (ICP) in an immunosuppressed host (Table 1). Diagnosis: lumbar puncture with opening pressure, CSF India ink stain, CrAg, and culture; manage elevated ICP with serial therapeutic punctures.

Induction: L-AmB 3–4 mg/kg/day + flucytosine 100 mg/kg/day for ≥14 days. In HIV/resource-limited settings: single-dose L-AmB 10 mg/kg + flucytosine + fluconazole 1200 mg/day for 14 days. If L-AmB is unavailable: ABLC or amphotericin B deoxycholate + flucytosine. If flucytosine is unavailable: available amphotericin formulation + fluconazole 1200 mg/day [33,72].

Consolidation/ART: fluconazole 400–800 mg/day for 8 weeks, then 200 mg/day for 12 months or until immune restoration (Table 6). Start antiretroviral therapy (ART) at 2–4 weeks if the patient has improved, ICP is controlled, and cultures are negative; otherwise defer 4–6 weeks [73].

## 6. Barriers and Opportunities in Latin America

**Figure 4:**
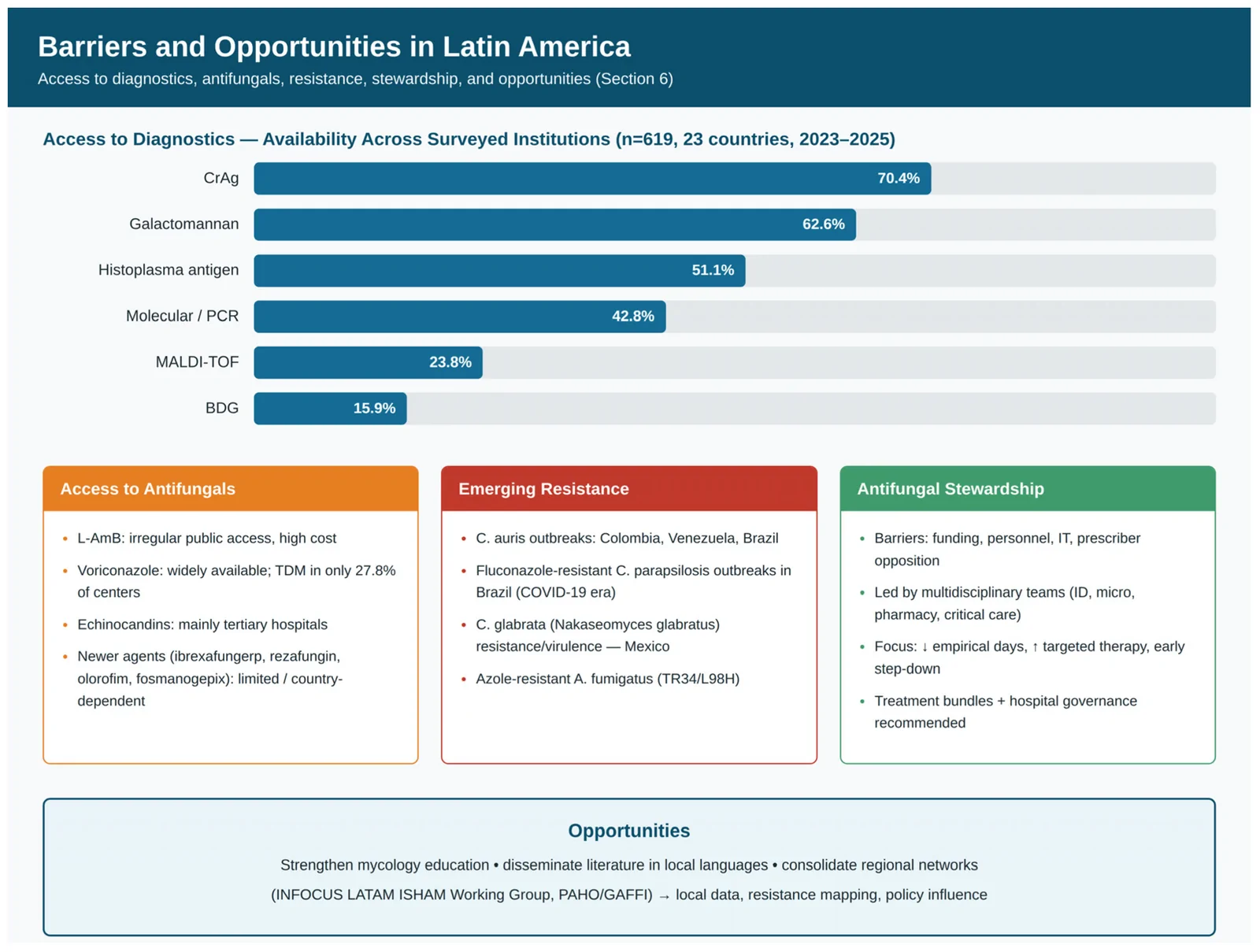
Barriers and opportunities for the management of invasive fungal disease in high-acuity hospitals across Latin America: access to diagnostics, access to antifungals, emerging antifungal resistance, antifungal stewardship, and opportunities (Section 6).

The management of IFDs in Latin America faces the challenge of controlling potentially lethal endemic mycoses and emerging opportunistic infections within health-care systems marked by unequal resources. Critical social determinants, such as migration, internal displacement, and gaps in access to care, increase the vulnerability of immunosuppressed patients and hinder epidemiologic follow-up. Recognizing these gaps is the first step toward optimizing care (Figure 4).

### 6.1. Access to Diagnostics

Fungal infections remain a neglected public health problem. An early regional survey of 129 centers in 24 countries, published in 2019, found that CrAg was the most available biomarker, with routine use reported in 75% of institutions at that time, followed by galactomannan in 48%; *Histoplasma* antigen and BDG were reported in only 22% and 17% of institutions, respectively, and rapid identification tools such as MALDI-TOF and fungal PCR were each available in less than 20% of institutions surveyed [74]. A more recent multinational survey of 619 institutions across 23 countries in Latin America and the Caribbean, conducted between 2023 and 2025, found a broadly similar pattern with modest gains: CrAg availability of 70.4%, galactomannan of 62.6%, *Histoplasma* antigen of 51.1%, molecular diagnostics (mainly for *Pneumocystis jirovecii* and *Aspergillus*) of 42.8%, and MALDI-TOF of 23.8%, while BDG remained the least available test at only 15.9% [75]. These persistent gaps represent an ongoing missed opportunity for early diagnosis and targeted therapy (Figure 4). These figures describe the institutions surveyed and should not be read as uniform coverage across the region: although some countries have high-acuity centers that meet international standards, diagnostic capacity remains concentrated in referral hubs, generating inequitable access even within the same country. In most hospitals, limited diagnostic availability perpetuates a model based on clinical suspicion, delayed cultures, and prolonged empirical treatment, with greater exposure to toxicity, costs, and additional ICU days.

### 6.2. Access to Antifungals

The regional therapeutic arsenal is similarly heterogeneous (Figure 4), and the same caveat about source-specific rather than region-wide representativeness applies. L-AmB has irregular availability in public systems, and its high cost limits sustained use, especially for prolonged courses or severe mold disease. Voriconazole is widely available, but its utility is affected by limited TDM—available in only 27.8% of surveyed centers across Latin America and the Caribbean [75]—exposing patients to hepatotoxicity, neurotoxicity, or subtherapeutic concentrations. This gap is clinically consequential: even where TDM is performed, a substantial proportion of voriconazole trough concentrations fall outside the therapeutic range, particularly in older patients and those with hypoalbuminemia, underscoring the value of expanding monitoring capacity in the region [76]. Echinocandins are usually available in tertiary hospitals, but access outside these referral centers is inconsistent and has not been systematically surveyed. Newer molecules, such as ibrexafungerp, rezafungin, olorofim, or fosmanogepix, still have limited or country-dependent registration and clinical availability.

### 6.3. Emerging Antifungal Resistance

The region is not immune to the global resistance crisis (Figure 4). The high burden of candidemia in Latin America amplifies the clinical impact of shifts in species distribution and antifungal susceptibility. In-hospital dissemination of multidrug-resistant *C. auris* has been documented, with severe outbreaks in Colombia, Venezuela, and Brazil [7,77]. A recent global meta-analysis of 81 studies published between 2015 and 2024 found fluconazole resistance in 92.5% of *C. auris* isolates, voriconazole resistance in 49.0%, and amphotericin B resistance in 51.0%, with mortality reaching 41.5% among immunocompromised patients—findings that underscore the need for stronger surveillance in low-resource regions [78]. In addition, selective pressure from broad azole and echinocandin use has been associated with resistant profiles in prevalent species. Interhospital outbreaks of fluconazole-resistant *Candida parapsilosis* were reported in Brazil during the COVID-19 era [79], along with growing interest in resistance and virulence mechanisms of *Candida glabrata*—currently *Nakaseomyces glabratus*—in Mexico [80]. Among molds, azole resistance in *Aspergillus fumigatus*, including environmental mutations such as TR34/L98H, represents an emerging regional threat [81]. These resistance phenotypes arise through interconnected mechanisms—including ERG11 and FKS1/FKS2 target-site mutations, efflux-pump overexpression, and biofilm-mediated drug tolerance—that are increasingly viewed as therapeutic targets in their own right, including candidate approaches such as fungal phosphoglucomutase inhibition [82,83].

### 6.4. Antifungal Stewardship (AFS)

The implementation of antifungal optimization programs in Latin America faces barriers that differ from those in high-income countries, including a critical lack of funding, personnel, information technology, and occasional prescriber opposition (Figure 4). In this context, AFS should be led by multidisciplinary teams, with active participation from infectious diseases, microbiology, pharmacy, and intensive care, adapting strategies to the actual availability of diagnostic tests and drugs [84]. Success metrics should pragmatically focus on reducing unnecessary days of empirical therapy, increasing the proportion of targeted therapy, promoting early oral step-down, and achieving de-escalation to fluconazole when microbiology and susceptibility results allow it. To maximize clinical impact and overcome regional diagnostic limitations, the implementation of clinical care pathways or “treatment bundles,” firmly supported by hospital governance, is strongly recommended as a core strategy [71,85].

### 6.5. Opportunities

Despite these challenges, clinical mycology in Latin America is growing (Figure 4). Strengthening medical mycology education from undergraduate training, disseminating scientific literature in local languages, and consolidating regional networks—such as the INFOCUS LATAM ISHAM Working Group and PAHO/GAFFI—offer a strategic platform to generate local data, map resistance, adapt diagnostic algorithms to available resources, and influence public health policy toward more equitable access to diagnosis and treatment [86].

## 7. Conclusions

Invasive fungal disease in Latin American high-acuity hospitals cannot be managed by directly transplanting algorithms built for high-resource settings. A workable pathway instead starts from a structured “window of suspicion” built on host risk factors, proceeds through a diagnostic work-up that is explicitly adapted to regional biomarker and molecular-testing availability, and resolves into syndrome-specific clinical pathways for candidemia, invasive aspergillosis, mucormycosis, and cryptococcal meningitis. Across these syndromes, the same principles recur: clinical prediction rules are most useful for ruling out disease rather than triggering therapy; imaging and biomarkers must be interpreted against host biology rather than a single universal cut-off; and early, syndrome-directed antifungal therapy—followed by prompt de-escalation once culture and susceptibility data allow it—represents, in our view, a practical framework for balancing mortality reduction against antifungal stewardship goals, rather than a demonstrated optimal strategy. Persistent gaps in diagnostic access, TDM, and newer antifungal availability remain the principal barriers to implementing these pathways consistently across the region. Strengthening regional networks, mycology training, and locally adapted stewardship programs represents the most realistic route toward earlier, better-targeted antifungal therapy in Latin American high-acuity hospitals.

## Figures and Tables

**Table 1:** Specific risk factors, clinical/radiological red flags, and immediate diagnostic action by fungal pathogen for rapid bedside identification (Section 2.5).

Pathogen	Host Risk Factors	Clinical & Radiological Red Flags	Immediate Diagnostic Action
*Candida* spp. (*Candida* Score/Ostrosky-Zeichner)	Broad-spectrum antibiotics ≥ 72 h; CVC and total parenteral nutrition; dialysis; high-risk abdominal surgery (GI perforation, anastomotic leak, necrotizing pancreatitis); ICU stay ≥4 days; multifocal *Candida* colonization; consider *C. auris* specifically if prior antifungal exposure, prolonged ICU stay, interfacility transfer, or known outbreak setting	Severe sepsis/shock without clear focus; intra-abdominal collection/secondary-tertiary peritonitis; persistent candidemia despite therapy; retinal lesions or hepatosplenic abscesses (post-neutropenia); disseminated (hematogenous) skin lesions	Serial blood cultures + serum BDG; rule-out: *Candida* Score < 3 or negative Ostrosky-Zeichner (a positive score alone should not trigger empirical therapy); if *C. auris* suspected: species-level ID + susceptibility testing + contact precautions
*Aspergillus* spp. (EORTC/MSGERC)	Neutropenia < 500/µL >10 days; hematologic malignancy/GVHD; allogeneic HSCT or SOT; corticosteroids ≥ 0.3 mg/kg/day for ≥3 weeks; severe viral pneumonia (COVID-19/influenza)	Dense nodule ± halo sign; air crescent sign or cavitation; wedge-shaped or lobar consolidation; tracheobronchitis (bronchoscopy)	Chest CT; serum and/or BAL galactomannan; PCR (if available)
*Mucorales* (ECMM)	Uncontrolled diabetes/DKA; hematologic malignancy/neutropenia/HSCT; iron overload/deferoxamine; trauma or severe burns; severe COVID-19 with corticosteroids	Necrotizing sinusitis + palatal/nasal eschar; proptosis or periorbital edema; reversed halo sign or >10 nodules on CT; necrotic skin lesions	Contrast MRI (rhino-orbital-cerebral) or chest CT; urgent tissue biopsy; do not delay management for cultures
*Cryptococcus* spp. (ECMM/ISHAM/ASM)	HIV with CD4 < 100/µL (screen if <200); SOT or hematologic malignancy; anti-CD20 or kinase inhibitors; eucalyptus exposure in immunocompetent hosts (*C. gattii*)	Subacute headache + fever; altered mental status/cranial nerve palsy; signs of intracranial hypertension; pulmonary nodules; umbilicated skin lesions	Serum CrAg; if neurological suspicion: lumbar puncture with opening pressure + CSF CrAg/culture
*Fusarium* spp./*Scedosporium-Lomentospora* (ESCMID/ECMM/ISHAM)	Similar to *Aspergillus* (severe neutropenia, HSCT, SOT—*Scedosporium*: especially lung transplant); near-drowning in polluted water (*Scedosporium*); cystic fibrosis	Multiple/necrotic target skin lesions (*Fusarium*); brain abscess(es) (*Scedosporium*); may mimic *Aspergillus* on chest imaging	Blood cultures (high yield for *Fusarium*, unlike *Aspergillus*); skin or CNS biopsy/culture; antifungal susceptibility testing
*Trichosporon* spp. (ECMM/ISHAM/ASM)	Hematologic malignancy with neutropenia; breakthrough infection during echinocandin prophylaxis/therapy; corticosteroids, broad-spectrum antibiotics, CVCs	Fungemia with skin lesions resembling disseminated candidiasis; funguria, renal involvement; chorioretinitis	Blood cultures + species identification (MALDI-TOF if available); serum CrAg may cross-react; confirm susceptibility (voriconazole preferred)
*Pneumocystis jirovecii* (ECIL)	HIV with CD4 < 200/µL; prednisone ≥ 20 mg/day for ≥4 weeks; alemtuzumab, fludarabine, or calcineurin inhibitors	Subacute progressive dyspnea + dry cough; hypoxemia disproportionate to exam/X-ray; CT: bilateral diffuse ground-glass opacities	Induced sputum or BAL with direct staining; serum BDG; does not grow in standard culture
*Histoplasma*/*Coccidioides* spp. (PAHO 2020; IDSA 2025)	HIV with CD4 < 200/µL (especially <150); SOT, anti-TNF, or other immunosuppression; *Coccidioides* also: pregnancy (3rd trimester), Black/Filipino ancestry, IL-12/IFN-γ or STAT3 defects	Prolonged fever + weight loss + cytopenias; hepatosplenomegaly or lymphadenopathy; reticulonodular/miliary infiltrates (may mimic tuberculosis); *Coccidioides* may disseminate to CNS, bone, or skin	Urine/serum antigen, fungal culture, histopathology, and serology per syndrome and availability; bone marrow biopsy/culture if disseminated
*Paracoccidioides* spp. (Latin American epidemiologic review)	Predominates in immunocompetent hosts; chronic form (>90%): rural male > 30 y, smoking/alcohol, prolonged occupational exposure; acute/juvenile form (<10%): children or young adults < 30 y, either sex	Chronic form: painful oral/mucosal lesions, cough, dyspnea, skin lesions; acute/juvenile form: generalized lymphadenopathy + hepatosplenomegaly + bone lesions; minimal lung involvement in the acute form	Direct exam (KOH): multiple budding “steering wheel” pattern is diagnostic; supportive serology; biopsy if uncertain
*Sporothrix* spp. (*S. schenckii/S. brasiliensis*)	Traumatic inoculation (soil, thorns, plant matter—classic, *S. schenckii*); cat scratch/bite exposure (zoonotic, especially Brazil/Argentina, *S. brasiliensis*); disseminated disease risk: advanced HIV, chronic alcoholism, diabetes	Lymphocutaneous pattern: nodule/ulcer at inoculation site + ascending nodules along lymphatic chain; disseminated: osteoarticular, ocular, or meningeal involvement; rare chronic cavitary pulmonary disease	Fungal culture of lesion/tissue (gold standard); adjunctive histopathology + panfungal PCR if available

Abbreviations: BAL, bronchoalveolar lavage; BDG, (1,3)-β-D-glucan; CD4, cluster of differentiation 4; CNS, central nervous system; CrAg, cryptococcal antigen; CSF, cerebrospinal fluid; CT, computed tomography; CVC, central venous catheter; DKA, diabetic ketoacidosis; GI, gastrointestinal; GVHD, graft-versus-host disease; HSCT, hematopoietic stem cell transplantation; ICU, intensive care unit; KOH, potassium hydroxide; MRI, magnetic resonance imaging; NPV, negative predictive value; PCR, polymerase chain reaction; SOT, solid organ transplantation.

**Table 5:** Diagnostic performance of galactomannan, β-D-glucan, and *Aspergillus* PCR for invasive aspergillosis, by host type (Section 5.2).

Test	Host Type	Sensitivity	Specificity	Notes	Reference
Galactomannan (serum)	HSCT/hematologic malignancy	70–82%	81–92%	Best-characterized population; pediatric HSCT cohort: 91.3%/81.7%	[56]
	SOT	21–86%	80–89%	Wide range reflects heterogeneity; meta-analysis concludes GM more useful in HSCT than SOT	[57]
	Non-classical/other at-risk	≤40%	Not well established	Markedly decreased sensitivity attributed to limited angio-invasion with preserved immunity	[58]
Galactomannan (BAL)	HSCT/hematologic malignancy	73–100%	68–92%	Meta-analyses suggest ~85% sens/90–95% spec overall; higher cutoff (ODI 1.0 vs. 0.5) improves specificity without losing sensitivity	[58]
	SOT (esp. lung transplant)	60–90%	90–96%	Comparable accuracy to hematologic patients—BAL closes much of the serum performance gap	[58]
	Non-classical/other at-risk	Limited data	Limited data	Not separately established	[58]
β-D-glucan (serum)	HSCT/hematologic malignancy	60–80%	≥90%	Two consecutive positives raise specificity to 95–99%; pediatric allo-HSCT: 80% sens but only 55% spec	[58,59]
	SOT/ICU	Reduced vs. HSCT	Specificity concerns raised	Performs worse than in hematologic malignancy; data described as mixed/heterogeneous	[58,60]
	Non-classical/other at-risk	Limited data	Limited data	Not separately established	[58]
*Aspergillus* PCR (serum)	Mixed neutropenic/non-neutropenic (HSCT 33%, hematologic malignancy 26%, heart/liver/kidney transplant ~26%)	71.7% overall (82.1% neutropenic vs. 62.5% non-neutropenic, ns)	98.7%	PPV 79.6% vs. GM’s 27% in same cohort, due to high GM false-positive rate in neutropenic patients	[61]
*Aspergillus* PCR (BAL)	Mixed hematological + non-hematological (incl. SOT)	88.6%	95.5%	Outperformed GM index (56.3%/97.6%) and mycology (63.6%/94.5%) in same 613-patient cohort	[62]

**Table 6:** Comparative properties of azoles commonly used in Latin America for the treatment of invasive fungal disease (Section 5.3).

Drug	Dosing	Spectrum	TDM	Key Interactions	Regional Availability
Fluconazole	Loading 800 mg (12 mg/kg), then 400 mg (6 mg/kg) daily, IV/PO	Most *Candida* (albicans, parapsilosis, tropicalis); no activity vs. *C. krusei*; variable/dose-dependent vs. *C. glabrata*; no mold activity	Not routinely recommended	CYP2C9/3A4 inhibitor—warfarin, sulfonylureas, phenytoin; mildest interaction profile of the four	Widely available, generic, low-cost across the region
Voriconazole	Loading 6 mg/kg IV q12h ×2 (day 1), then 4 mg/kg IV q12h; oral 200–300 mg q12h (weight-based)	*Candida* + *Aspergillus* + other molds (*Fusarium*, *Scedosporium*); first-line for IA	Mandatory—target trough 1–5.5 mg/L (2–5.5 for *A. flavus/niger*); hepatotoxicity risk > 3–3.75 mg/L; highly variable (CYP2C19 polymorphism)	Strong CYP3A4 inhibitor—raises tacrolimus/cyclosporine; sirolimus contraindicated; rifampin/rifabutin/carbamazepine contraindicated; potentiates warfarin	Available across most of the region; TDM capacity is the inconsistent element
Posaconazole	300 mg BID day 1, then 300 mg once daily (tablet/IV); older suspension needs food/acidic environment	*Candida* + *Aspergillus* + *Mucorales*; used for mucormycosis and refractory/salvage IA	Recommended—target trough >1 mg/L (treatment), >0.7 mg/L (prophylaxis); hepatotoxicity risk > 3–3.75 mg/L	CYP3A4 inhibitor—raises tacrolimus and especially sirolimus levels; affects cyclosporine	More limited and costlier than fluconazole/voriconazole; tablet availability variable by country
Isavuconazole	Loading 372 mg isavuconazonium sulfate (=200 mg isavuconazole) IV/PO q8h ×6 doses (48 h), then 200 mg once daily	*Candida* + *Aspergillus* + *Mucorales*; approved for both IA and mucormycosis	Utility still debated; suggested range ~2–4 mg/L; low troughs reported in critically ill/ECMO patients	Less interaction with calcineurin inhibitors than voriconazole/posaconazole; contraindicated with strong CYP3A4 inducers/inhibitors; shortens QTc	Least available and most costly; more limited access than voriconazole

## Data Availability

No new data were created or analyzed in this study. Data sharing is not applicable to this article.

## Abbreviations

The following abbreviations are used in this manuscript:

IFD	Invasive fungal disease
IA	Invasive aspergillosis
CAPA	COVID-19-associated pulmonary aspergillosis
IAPA	Influenza-associated pulmonary aspergillosis
BDG	(1,3)-β-D-glucan
GM	Galactomannan
ODI	Optical density index
CrAg	Cryptococcal antigen
LFA/LFD	Lateral-flow assay/device
PCR	Polymerase chain reaction
mNGS	Metagenomic next-generation sequencing
BAL	Bronchoalveolar lavage
CT	Computed tomography
MRI	Magnetic resonance imaging
CSF	Cerebrospinal fluid
TDM	Therapeutic drug monitoring
HSCT	Hematopoietic stem cell transplantation
SOT	Solid organ transplantation
GVHD	Graft-versus-host disease
ICU	Intensive care unit
CVC	Central venous catheter
TPN	Total parenteral nutrition
RRT	Renal replacement therapy
DKA	Diabetic ketoacidosis
ICP	Intracranial pressure
ART	Antiretroviral therapy
NPV/PPV	Negative/positive predictive value
MIC	Minimum inhibitory concentration
PK/PD	Pharmacokinetic/pharmacodynamic
L-AmB	Liposomal amphotericin B
ABLC	Amphotericin B lipid complex
AFS	Antifungal stewardship
EORTC/MSGERC	European Organization for Research and Treatment of Cancer/Mycoses Study Group Education and Research Consortium
ECMM/ISHAM/ASM	European Confederation of Medical Mycology/International Society for Human and Animal Mycology/American Society for Microbiology
FUNDICU	Invasive Fungal Diseases in Adult Patients in Intensive Care Unit (consensus definitions)
AspICU	Clinical algorithm to diagnose invasive pulmonary aspergillosis in critically ill patients
EBCS	Evidence-based clinical standard
MALDI-TOF	Matrix-assisted laser desorption/ionization time-of-flight (mass spectrometry)
